# Pixel-Wise Crack Detection Using Deep Local Pattern Predictor for Robot Application

**DOI:** 10.3390/s18093042

**Published:** 2018-09-11

**Authors:** Yundong Li, Hongguang Li, Hongren Wang

**Affiliations:** 1 School of Electronic and Information Engineering, North China University of Technology, Beijing 100144, China; liyundong@ncut.edu.cn; 2 Unmanned Systems Research Institute, Beihang University, Beijing 100191, China; 3 School of Electronic and Information Engineering, Beihang University, Beijing 100191, China; wanghongren616@hotmail.com

**Keywords:** local pattern predictor, crack detection, bridge inspection, convolutional neural networks, robotic vision

## Abstract

Robotic vision-based crack detection in concrete bridges is an essential task to preserve these assets and their safety. The conventional human visual inspection method is time consuming and cost inefficient. In this paper, we propose a robust algorithm to detect cracks in a pixel-wise manner from real concrete surface images. In practice, crack detection remains challenging in the following aspects: (1) detection performance is disturbed by noises and clutters of environment; and (2) the requirement of high pixel-wise accuracy is difficult to obtain. To address these limitations, three steps are considered in the proposed scheme. First, a local pattern predictor (LPP) is constructed using convolutional neural networks (CNN), which can extract discriminative features of images. Second, each pixel is efficiently classified into crack categories or non-crack categories by LPP, using as context a patch centered on the pixel. Lastly, the output of CNN—i.e., confidence map—is post-processed to obtain the crack areas. We evaluate the proposed algorithm on samples captured from several concrete bridges. The experimental results demonstrate the good performance of the proposed method.

## 1. Introduction

In robot applications, bridge inspection is extremely necessary to set up strategies of bridge repair and rehabilitation, especially when maintenance resources are limited. Cracks are the most important representations of the health condition of concrete structures. It is reported that more than 100,000 bridges suffer from cracking in the United States [1]. Therefore, the evaluation of cracking plays a vital role in bridge inspection. The traditional crack detection relies on human visual inspection, which is time-consuming and labor intensive when examining a large span bridge. To address these limitations, computer-vision based method is promising to enhance human visual inspection or to be combined with mechanical and aerospace structures, such as aerial robots [2], to conduct a robotic crack inspection for crack detection. 

Conventional computer-vision based methods for crack detection proceed in a two-phase fashion: feature extraction and feature classification. The key issue lies in the process of designing distinguishing features. Basically, feature design principles may be divided into two groups: handcrafted methods and learned methods. In the past decades, the handcrafted features, such as histograms of oriented gradients (HOG) [3] and scale-invariant feature transform (SIFT) [4], had made great achievements in face recognition, pedestrian detection, vision inspection, and so on. However, these are very rudimentary methods in the form of visual inspection which have several disadvantages, including limited accuracy and narrow vision field of the whole bridge deck. Additionally, it is a dangerous task for humans to conduct visual crack inspection, especially with passing traffic [5]. As a result, a robotic system where robots are incorporated with visual detection methods to implement crack assessment has large development prospects. Despite being widely used, the existing robotic systems still employ manual approaches mentioned above for feature analysis, which leaves the problem of low accuracy unsolved. Recently, it has been demonstrated that the hierarchical features extracted with deep learning can outperform handcrafted features in most cases [6]. Crack detection based on deep learning becomes a novel and potential approach to research.

## 2. Related Works

Robotic systems incorporated with visual techniques to solve problems is no longer a new topic in the field of computer vision, including visual perception, object recognition, detection, and tracking. A human-robot interactive framework was proposed to endow robots with the ability to localize a person by processing visual and audio data [7], so that people can be directed attention to whom they were interacting. Robotic application is also extensively developed in 3D visual problems. Amatya et al. developed a method of locating shaking positions based on objects pixel locations in the images [8]. An enhanced CHT [9] is employed for estimating the trajectory of a spherical target in three dimensions to improve tracking accuracy. For robotic navigation in unstructured environments, a method based on ToF cameras [10] was provided for 3D obstacle detection and classification. Meanwhile, in the area of industrial and daily application [11], the robot mechanism presents a promising prospect. A real-time collision detection algorithm for marine robotic manipulation was presented to be a useful pilot assisting tool for subsea intervention operations [12]. In addition, Leite et al. proposed a new skill-based architecture for MR which is used to overcome the difficulties in current autonomous robot design [13] to improve robotic performance.

Many crack detection algorithms have been developed before now. Koch et al. summarized the computer-vision based methods, and roughly categorized them into pre-processing, feature-based, model-based, pattern-based, and 3D reconstruction types [14]. Herein, we divide the existing algorithms into non-learning-based and learning-based from the perspective of feature representation and classification methods. 

Most of the non-learning-based methods employ image processing technologies, such as noise filtering, edge detection, binary thresholding, and morphological operations [15,16,17,18,19]. Adhikari et al. proposed a crack detection method by combining histogram enhancement, median filtering, and thresholding [20]. Images are enhanced and smoothed by histogram equalization and median filter first, then the candidate cracks are obtained by subtracting smoothed images from original images. Dinh et al. proposed a histogram thresholding-based method [21]. Moving average filters were employed to remove noise from images, then images were segmented with threshold determined using histogram peak and valley information. However, this method can only be used in situations of high contrast images. Lim et al. presented an automatic inspection system using mobile robotic equipped with cameras [22]. A Laplacian of Gaussian (LoG) algorithm was utilized in their scheme. Yamaguchi et al. proposed a crack detection method based on percolation image processing technique [23]. In 2014, Nguyen et al. proposed a two-stage detection method [24]. Images were enhanced by phase symmetry-based filters first, then binary images were obtained using thresholding and morphological operations. In the second stage, the center line of a crack was fitted by cubic splines and crack edges were determined by pixel intensity perpendicular to the splines. Kapela et al. 2015 proposed a HOG based crack detection scheme which was used for pavement inspection [25]. Although there are many studies, the bulk of non-learning-based approaches use only low-level image processing techniques, which easily fail to separate cracks from complicated backgrounds because they are noise sensitive, and the surface images always suffer from dirt, shadows, and other factors causing noise [1]. Furthermore, the binary segmentation with a fixed threshold in non-learning-based methods is lacking flexibility with respect to variations of environments.

Compared with non-learning methods, learning-based approaches learn patterns from surface images and use patterns instead of pixels to predict cracks, which can alleviate negative effects of noise. The potentials of machine learning for crack detection—such as support vector machines (SVM) [26], neural networks [27], and principal component analysis (PCA)—were explored by researchers [28]. Lattanzi et al. presented a crack detection method using Candy operator and K-means clustering algorithm [29]. In 2015, Bu et al. proposed an automatic crack detection scheme [30] where wavelet features were firstly extracted using a sliding window texture analysis technique, then features were classified by SVM. Prasanna et al. presented an automatic crack detection algorithm, called spatially tuned robust multifeature (STRUM) classifier [1]. In their scheme, images were tiled as patches, and cracks of each patch were approximated by curve fitting using random sample consensus (RANSAC) algorithm. To eliminate parameter adjustment, several popular classifiers—such as SVM, AdaBoost, and random forest—were used to remove false fitting results. In 2017, Chaudhury et al. proposed a spatial-temporal non-linear filtering method combined with conditional random fields (CRF) [31]. Experimental results showed that their method can obtain high accuracy. Li et al. 2017 developed a region-based active contour model combined with Canny operator for concrete crack segmentation, and SVM was used to eliminate noises [32]. Sparse autoencoder (SAE) and tensor voting were introduced in a pavement crack inspection by Qian et al. [33]. Features of potential patches were extracted by SAE, and then classified by a softmax classifier. In 2016, Schmugge et al. proposed a deep neural network-based method which was used in crack detection for nuclear power plant [34]. Both training and testing images were divided into patches of 224 × 224 pixels, and a GoogleNet was used to classify each patch into crack or non-crack objects. Both methods in [33,34] can only get rougher detection results because it was block-wise and could not locate cracks on a pixel level. 

As discussed above, on the basis of the existing research, crack detection algorithms still face two challenges: strong adaptability to noisy environment and pixel-wise high accuracy requirement.

## 3. Work in This Paper

In this study, a completely different solution for crack detection is explored. The idea was motivated by the fact that adjacent pixels are highly spatially correlated. Whether a pixel belongs to a crack depends upon its surroundings. Basically, pixels of cracks are located on regular geometrical lines, while pixels of noise are not. Therefore, this distribution pattern could facilitate crack identification. The crucial point is how to capture this distribution pattern. In our scheme, CNN is employed to extract abstract representation of pixels’ pattern, then each pixel is classified into crack or non-crack using the patterns as inputs of CNN classifier. Experiments show that patterns extracted by CNN are robust enough to noise and variation of environments. 

This research has three main contributions. (1) We provide a local pattern predictor (LPP) which reveals an underlying mapping function existed between a pixel probability to belong to cracks and its context. (2) A CNN is designed to learn this mapping function of LPP in our scheme, which is used to predict the probability of each pixel according to its context. To the authors’ best knowledge, this represents a first attempt to use deep learning to forge an LPP and detect cracks in a pixel-wise way. (3) A typical database, including concrete crack images, is collected and open to fill the blank of the database in the area of crack detection. The image samples are available at https://github.com/VivianWang0616/CrackDetectDLPP/tree/master.

## 4. Proposed Method

### 4.1. Scheme of Proposed Method

In this paper, we propose a pixel-wise based method leveraging patterns of each pixel. In other word, each pixel’s probability belongs to cracks is predicted using a local pattern predictor according to its pattern. The local pattern predictor is constructed based on CNN. It can divide each pixel into crack or non-crack, using as context a patch centered that pixel.

**Figure 1:**
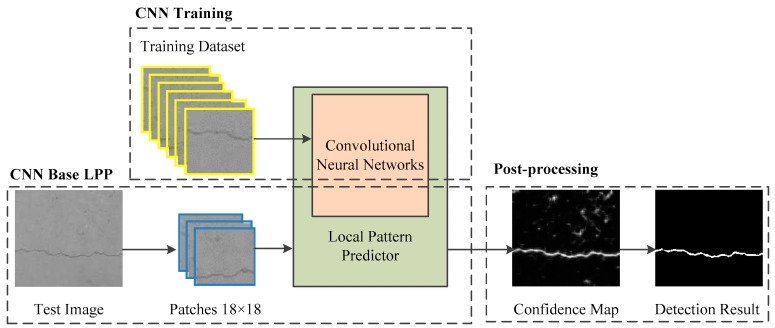
Overview of the proposed algorithm.

The overall diagram of the method is shown in Figure 1. In the scheme, a seven-layer CNN is customized to facilitate the crack classification task. It is trained by a training dataset collected from different bridges. Subsequently, the well-trained CNN is used as a basis to construct a local pattern predictor. An 18 × 18 patch centering each pixel tiled from concrete surface images is fed into local pattern predictor. Thus, the confidence map of the test image is obtained from the output of LPP. Finally, the confidence map is post-processed to locate crack areas.

### 4.2. Local Pattern Predictor

As mentioned before, learning-based methods always divide test images into small patches, and features extracted from the patches are compared with those of reference images to identify the patches which containing cracks. These kinds of methods can only label the blocks contain cracks, but cannot locate the crack pixels accurately. In this paper, we take a different approach, by predicting the probability of each pixel according to its context, which is called local pattern predictor (LPP).

The context of a pixel is defined as a rectangular area centered at this pixel, with the width of *w* and the height of *h*. Whether a pixel belongs to a cracked area is related to its context because the concrete surface image has a strong 2D local structure. Spatially adjacent pixels are always highly correlated. The grayscale values of pixels in the centering rectangle are arranged to construct a local pattern. Let $q_{i}$ be the vector of the local pattern of the central *i*-th pixel, and $p_{i}$ be the probability of the central *i*-th pixel to belong to a cracked area, then the mapping between and can be expressed as

(1) $$f(q_{i})=p_{i}$$

The key point is to find a non-linear mapping to approximate the relationship presented by Equation (1). In this paper, CNN is trained to learn this mapping from a large set of samples. Studies have shown that the response of the human brain cortex to external visual stimulus is layered. The cortex responds to line information, such as the edges of objects, and then perceives shape information and the objects themselves. Feature representation of a CNN is abstracted layer by layer, which conforms to the learning process of human brain. It also has a capability of shift, scale, and distortion invariance. These merits make CNN well-suited to be a basis of local pattern predictor and to deal with degraded image quality affected by noise, variation of illumination, angles, and positions of cameras.

### 4.3. Convolutional Neural Networks for LPP

Since Hinton [35,36] proposed a greedy layer-wise pre-training algorithm to initialize the weights of deep architectures, artificial neural networks have been revived. Deep neural networks have become a new popular topic and advanced in image classification, object tracking [37] and recognition [38], gesture recognition [39], action recognition [40], defect inspection [41,42,43,44], voice recognition, natural language understanding, etc. Popular deep learning frameworks include stacked autoencoders, convolutional neural networks, and restricted Boltzmann machine.

**Figure 2:**
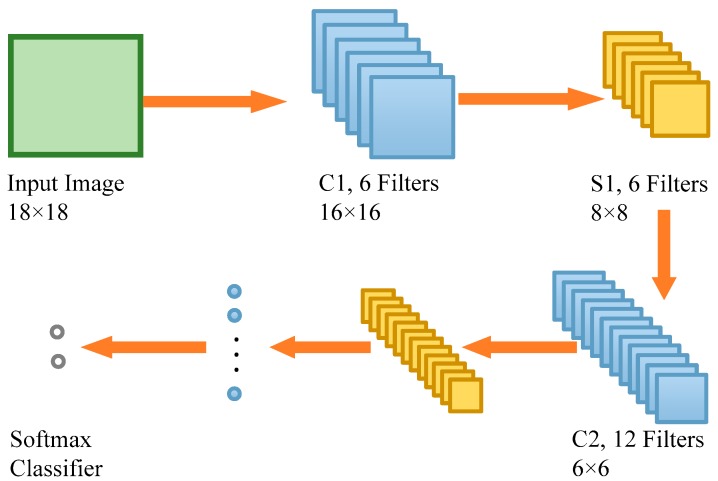
Architecture of CNN used in the proposed method.

In the proposed method, a seven-layer CNN based on the LeNet-5 [45] was designed (Figure 2). The input is an 18 × 18 image, which is corresponding to the size of a patch centering each pixel. Layer C1 is a convolutional layer with six feature maps. Each point of C1 feature maps is connected to a 3 × 3 neighborhood in the input image by a 3 × 3 convolutional filter. Consequently, the size of each map of layer C1 is 16 × 16. There are totally 60 trainable parameters and 15,360 connections in layer C1.

Layer S1 is a sub-sampling layer with six feature maps. The size of each map of layer S1 is 8 × 8 because each unit in S1 is corresponding to a 2 × 2 neighborhood in layer C1 using a max-pooling technique. The meaning of max-pooling is to choose a maximum within the 2 × 2 neighborhood. Layer C2 is a convolutional layer with 12 feature maps. Each unit of C2 feature maps is connected to a 3 × 3 neighborhood in the layer S1 by a 3 × 3 convolutional filter. The size of each map of layer C2 is 6 × 6. There are totally 120 trainable parameters and 25,920 connections in layer C2. Layer S2 is also a sub-sampling layer with 12 feature maps. The size of each map of layer S2 is 3 × 3 because each unit in S2 is corresponding to a 2 × 2 neighborhood in layer C2 using a max-pooling technique. Layer C3 is still a convolutional layer with 54 feature maps. Since the size of each map in layer S2 is 3 × 3, the size of feature map of layer C3 is 1 × 1 after a 3 × 3 convolutional calculation. Layer C3 can be treated as a full connection layer with 54 hidden units. The output of layer C3 is a 1 × 54 vector, which is called feature vector of the input image extracted by CNN. There are totally 540 trainable parameters and 6480 connections in layer C3. The last layer is a softmax classifier consisting of two units, which divides each feature vector into crack or non-crack categories. 

To train the seven-layer CNN, a training dataset comprised of 326,000 samples were collected from 45 images of different bridges, which includes 56,000 positive samples and 270,000 negative samples. The training parameters are presented in Section 5. 

### 4.4. Post-Processing

Confidence map is generated when a test image is fed into LPP, which contains each pixel’s probability, indicating whether it belongs to crack or non-crack. Subsequently, post-processing is conducted to segment the confidence map with a fixed threshold, and isolated noisy points are removed to obtain detection results. The binary threshold is simply fixed to 0.5.

## 5. Experimental Results

### 5.1. Data Set

**Figure 3:**
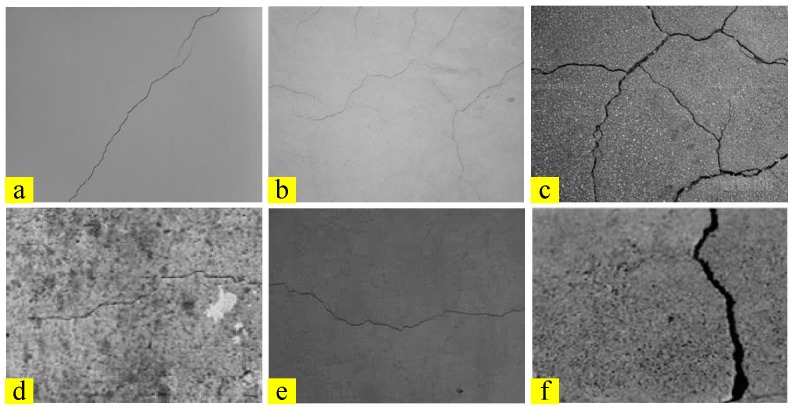
Examples of crack image: (**a**) clear and standard; (**b**) weak and disorderly; (**c**) noise; (**d**) dirty and blurred; (**e**) dark; (**f**) wide crack.

A data set is provided using images of real bridge cracks under different imaging environments and different bridge surfaces. Some challenging factors exist in the data set, which are illustrated in Figure 3. Figure 3a is a clear and distinct standard crack image. In Figure 3b, cracks are weak and disorderly. In Figure 3c, background noise is strong. In Figure 3d, the background is dirty and blurred. In Figure 3e, the illumination is dark. In Figure 3f, the crack is wide.

In our study, all the images are divided into two parts: 45 images for training and 5 for testing. Based on the 45 training images, 326,000 patches are sampled, which are used to train a seven-layer CNN. Among these samples, 56,000 patches are positive samples which contain cracks, and the other 270,000 are negative samples chosen from the non-crack background. We will discuss how to sample these patches later in Section 6.

### 5.2. Metrics

**Figure 4:**
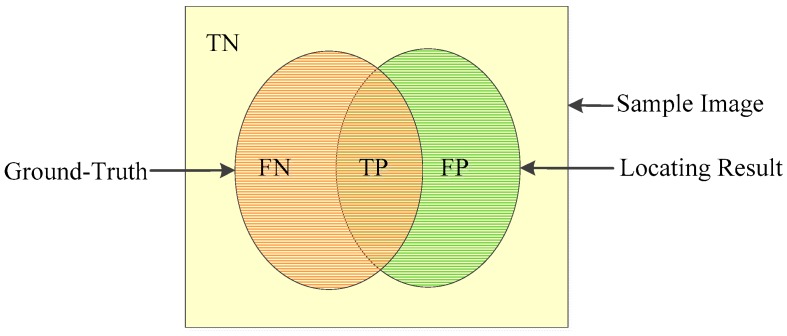
Definitions of TN, FN, TP, and FP.

A group of metrics including accuracy, recall and precision were employed to quantify the classification accuracy. The definition of accuracy, recall, and precision are described as Equations (2)–(4), where true positive (TP), true negative (TN), false positive (FP), and false negative (FN) are labeled in Figure 4 [38].

(2) accuracy=(TP+TN)/(TP+TN+FP+FN)

(3) recall=TP/(TP+FN)

(4) precision=TP/(TP+FP)

### 5.3. Performance Comparisons

To evaluate the performance of the proposed LPP method, we compared it with two up-to-date algorithms: STRUM proposed by Prasanna et al. [1] and block-wise CNN method used by Schmugge [34]. The testing code was implemented under Pytorch (version 0.3.0) and MATLAB (version R2012b). The testing computer was configured with Intel i7 processor with 3.2 GHz frequency, 64 GB memory and GPU GT730.

The architecture of CNN utilized in this paper is described in Section 3. Herein, the training parameters are presented as follows: learning rate is set to 0.001, momentum is 0.9, batch size is 100, and ReLU is used as activate function. We also implemented a CNN using sigmoid activate function. Experimental results demonstrated that ReLU performances are much better than sigmoid function.

**Figure 5:**
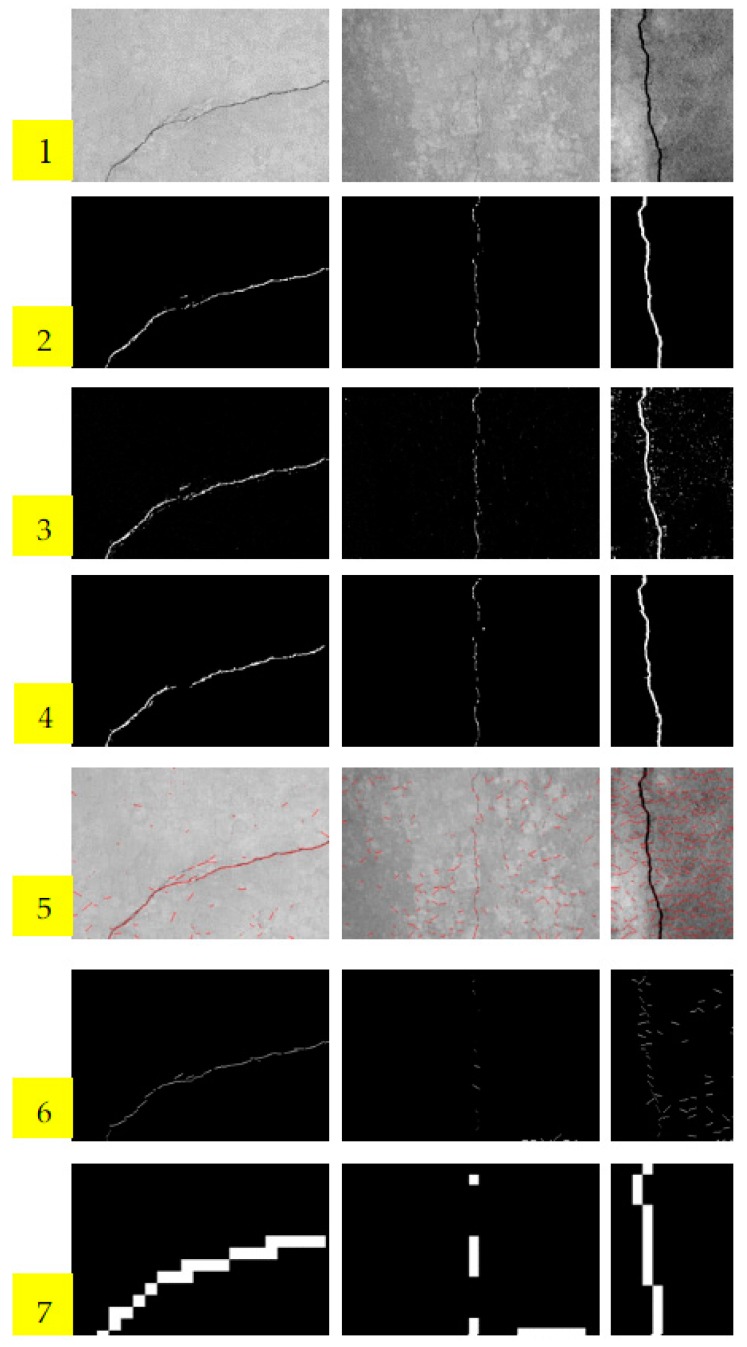
Crack locating results comparison. Row 1: Concrete surface images. Row 2: The ground-truths labeled manually. Row 3: Confidence maps of LPP method. Row 4: Results of LPP method after post-processing. Row 5: Line fitting results of STRUM method. Row 6: Results of STRUM method using AdaBoost classification. Row 7: Results of block-wise CNN method.

Parts of the experimental results are listed in Figure 5. The first row of Figure 5 is the original concrete surface images, which were contaminated by noise and clutters. It is difficult to separate cracks from background using traditional image processing technique in such scenarios. The second row is the ground truths labeled manually. The third row is the confidence maps predicted by LPP, and the fourth row is the final detection results of the proposed LPP method after post-processing. The rows 5 and 6 are the results of STRUM method. Row 5 gives the line fitness results using RANSAC algorithm. After fitness, patches including lines are classified by SVM, AdaBoost, and random forest in the literature [1]. The classification results of AdaBoost are only listed here because AdaBoost is superior to SVM in our experiments. We can see that STRUM method is effective only for the example of the left column. It is apparent that STRUM method is more suitable for thin cracks, and will fail when cracks are thick. It will also fail when test image is seriously contaminated, for example, the test image in the middle column. Conversely, LPP performs well in such scenarios. The last row shows the detection results of block-wise method using CNN. In the literature, Schmugge et al. [34] used LeNet architecture with input image of 224 × 224 pixels. Since the 224 × 224 block is too big for crack detection, we use a smaller block of 18 × 18 pixels instead. The architecture of CNN used in block-wise method is same as that of LPP method. Obviously, the detection results of block-wise CNN method are much rougher than the proposed LPP method.

The locating accuracies are listed in Table 1, and the best results are marked in bold. LPP gets the highest accuracy scores in all testing items. According to the analyses above, the LPP method appears superior to the STRUM and block-wise CNN methods in locating accuracy.

## 6. Discussion

### 6.1. Principle of Training Samples Choosing

**Figure 6:**
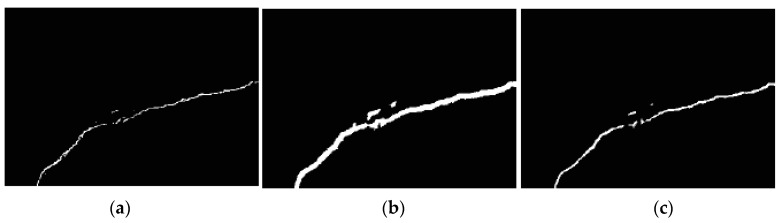
Crack-locating results compared of different sampling processes: (**a**) The ground-truths labeled manually; (**b**) detection result of uniform sampling; (**c**) detection result of uniform sampling with nearest neighbors.

**Figure 7:**
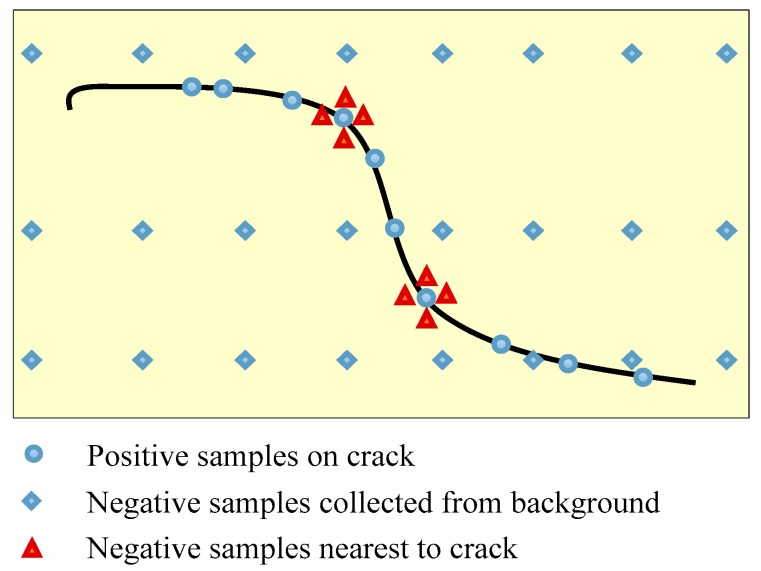
Illustration of training dataset sampling processes.

Training dataset is crucial to the performance of deep neural networks, especially for vision defect detection because there are many more backgrounds than defect pixels in the images. Positive samples are collected from all the pixels in crack areas. However, the negative sample selecting rule is much more skillful. In the preliminary experiments, training dataset is composed of positive samples collected from crack and negative samples selected uniformly from the background. The detection results using CNN trained by such training dataset are shown in Figure 6b. It is obvious that the detected cracks are much thicker than the ground truths. The reason is that points near to cracks are incorrectly categorized as cracks by the local pattern predictor. A uniform sampling method with nearest neighbors is proposed to address this issue in this paper, which is shown in Figure 7. Besides points sampled uniformly from background, points nearest to cracks are counted as negative samples. The detection results are greatly improved when using uniform sampling with nearest neighbors, which is shown in Figure 6c. The locating accuracies of different sampling processes are listed in Table 2.

### 6.2. Performance Improvement when Dataset Is Limited

As mentioned before, the numbers of positive and negative samples are imbalanced because most parts of concrete surface images represent backgrounds. This situation is common in visual inspection. To address the issue, we proposed a Fisher criterion based stacked denoising autoencoder (FCSDA) to enhance feature discrimination when only limited positive samples are available [38]. A preliminary study is conducted to bring the idea into CNN in this paper.

Suppose samples are divided into *L* classes, each class has $m_{i}$. Samples, i=1,2,…,L. $J_{\mathrm{int}ra}$ and $J_{\mathrm{int}er}$ are the intra-class and inter-class distance of features, which are defined as (5) and (6) respectively.

(5) $$J_{\mathrm{int}ra}=\frac{1}{2}\sum_{i}^{L}\sum_{k=1}^{m_{i}}{\left\|h_{w,b}\left(x\right)-M^{\left(i\right)}\right\|}^{2}=\frac{1}{2}\sum_{i}^{L}\sum_{k=1}^{m_{i}}\sum_{j=1}^{s}{\left({a_{j}}^{\left(k,n_{l}\right)}-{M_{j}}^{\left(i\right)}\right)}^{2}$$

(6) $$J_{\mathrm{int}er}=\frac{1}{2}\sum_{i=1}^{L}\sum_{j=i+1}^{L}{\left\|M^{\left(i\right)}-M^{\left(j\right)}\right\|}^{2}$$

where $h_{w,b}\left(x\right)$ is the feature vector extracted from input x, $n_{l}$ indicates the output layer of CNN, ${a_{j}}^{\left(k,n_{l}\right)}=f\left({z_{j}}^{\left(k,n_{l}\right)}\right)$ is the j-th element of the $n_{l}$ layer feature. *M*^(*i*)^ is the average feature of the i-th class, which is defined as

(7) $$M^{\left(i\right)}=\frac{\sum_{k=1}^{m_{i}}a^{\left(k,n_{l}\right)}}{m_{i}},i=1,2\ldots ,L$$

Add Fisher criterion term into a loss function of CNN, and loss function can be rewritten as

(8) $$J_{\left(w,b\right)}=\frac{1}{n}\sum_{i=1}^{n}\left(\frac{1}{2}{\left\|h_{w,b}\left(x^{\left(i\right)}-y^{\left(i\right)}\right)\right\|}^{2}\right)+\lambda \frac{J_{\mathrm{int}ra}}{J_{\mathrm{int}er}}$$

where $\frac{J_{\mathrm{int}ra}}{J_{\mathrm{int}er}}$ is the Fisher criterion in the feature space, λ is a ratio. Minimizing loss function will shorten the intra-class distance, while increasing the inter-class distance, which makes input patches easier to classify.

In this experiment, a reduced training dataset sub-sampled from the normal dataset is used to train CNN. It includes 20,000 positive samples and 140,000 negative samples. All the other parameters are the same as that in Section 4 except the fisher ratio set to 0.01. The experimental results comparison is listed in Table 3. Among it, CNN refers to a normal CNN trained by a reduced dataset, and Fisher-based CNN indicates Fisher criterion is added into the loss function of CNN. We can see that the performance of Fisher-based CNN is superior to normal CNN from the preliminary experiment. It is a worthy study direction to exploit the potentiality of Fisher criterion in CNN future applications.

### 6.3. Computational Time Analysis

The training and testing time of three methods are shown in Table 4. The testing time is counted from implementation of test image No. 1. Compared with the traditional Method 1, the training processed of CNN based Methods 2 and 3 are time-consuming. However, in training epoch, the proposed LPP is the lowest.

In application, the training time of LPP can be greatly shortened by using a GPU accelerator. Also, the training phase can be implemented offline. In that case, the proposed method can be employed for real-time inspection application. 

## 7. Conclusions

In this paper, we propose a robotic vision-based crack detection method. A local pattern predictor with the objective of detecting concrete surface cracks pixel-wisely can set up the underlying mapping function between a pixel’s probability of belonging to cracks and its context. Based on a typical CNN, the local pattern predictor can be trained used to detect cracks in images. A typical database, including concrete crack images, is collected and open to fill the blank of the database in crack detection. To the authors’ best knowledge, this represents a first attempt to use deep learning to forge a LPP and detect cracks in a pixel-wise way. In addition, training dataset selection principles are also investigated and a uniform sampling method with nearest neighbors is proposed. 

The experimental results show the proposed method appears effective and robust. In the future, authors would like to explore the potential of the Fisher criterion in CNN, as well as integrating the proposed method into an automatic inspection system. 

## Figures and Tables

**Table 1:** Crack locating accuracy comparison (the best results are marked in bold).

Test Images	Method	Acc (%)	Recall	Precision
No. 1	STRUM+AdaBoost	99.05	24.87	**83.19**
	Block-wise CNN	93.87	**86.97**	14.73
	LPP	**99.67**	84.17	73.38
No. 2	STRUM+AdaBoost	97.91	13.61	78.42
	Block-wise CNN	95.48	58.59	27.56
	LPP	**99.52**	**78.83**	**79.86**
No. 3	STRUM+AdaBoost	96.64	14.75	48.03
	Block-wise CNN	90.88	56.90	15.94
	LPP	**99.15**	**83.15**	**49.20**
No. 4	STRUM+AdaBoost	99.38	3.24	25.00
	Block-wise CNN	97.17	30.01	6.81
	LPP	**99.90**	**74.78**	**82.89**
No. 5	STRUM+AdaBoost	97.08	8.20	20.13
	Block-wise CNN	93.67	**93.42**	26.22
	LPP	**99.64**	89.99	**95.17**

**Table 2:** Accuracy of different sampling progress (the best results are marked in bold).

Test Images	Sampling Processes	Acc (%)	Recall	Precision
No. 1	Uniform	97.83	**95.40**	34.89
	Nearest neighbors	**99.67**	84.17	**73.38**
No. 2	Uniform	97.58	**80.19**	48.59
	Nearest neighbors	**99.52**	78.83	**79.86**
No. 3	Uniform	96.72	**85.52**	**50.40**
	Nearest neighbors	**99.15**	83.15	49.20
No. 4	Uniform	99.17	47.90	34.91
	Nearest neighbors	**99.90**	**74.78**	**82.89**
No. 5	Uniform	96.42	**99.93**	39.64
	Nearest neighbors	**99.64**	89.99	**95.17**

**Table 3:** Accuracy improved by Fisher criterion (the best results are marked in bold).

Test Images	Methods	Acc (%)	Recall	Precision
No. 1	CNN	99.27	49.74	**81.68**
	Fisher-based CNN	**99.29**	**53.83**	80.20
No. 2	CNN	98.34	31.51	**91.05**
	Fisher-based CNN	**98.40**	**35.47**	88.90
No. 3	CNN	96.84	5.79	76.92
	Fisher-based CNN	**97.02**	**12.79**	**83.33**
No. 4	CNN	99.37	2.88	22.22
	Fisher-based CNN	**99.69**	**5.74**	**34.78**
No. 5	CNN	99.01	93.10	72.66
	Fisher-based CNN	**99.05**	**93.23**	**73.40**

**Table 4:** Time spent comparison.

Index	Methods	Training Epoch	Training Time (s)	Testing Time (s)
1	STRUM AdaBoost	1000	138	5.1
2	Block-wise CNN	600	33,084	0.2
3	LPP	200	15411	10.7
